# A Case of Tizanidine Withdrawal and Road to Recovery

**DOI:** 10.7759/cureus.53444

**Published:** 2024-02-02

**Authors:** Tomas Escobar Gil, Kevin J McGeorge, Aaron J Jones

**Affiliations:** 1 Internal Medicine, University of New Mexico School of Medicine, Albuquerque, USA

**Keywords:** phenobarbital therapy, addiction, pain, psychiatry, neurology, internal medicine, withdrawal, overdose, tizanidine, toxicology

## Abstract

This case report highlights the complexities of tizanidine withdrawal in a 68-year-old woman with chronic pain. Tizanidine, a widely used imidazole-derived muscle relaxant, poses challenges due to the absence of standardized withdrawal protocols. The patient's presentation included hypertension and tachycardia following a gradual reduction in her outpatient tizanidine dose.

During the de-escalation of tizanidine, the patient experienced withdrawal symptoms, including severe body aches, hypertension, and tachycardia. Management during withdrawal involved a unique approach using a one-time dose of phenobarbital, a measure that allowed the resolution of hemodynamic instability and pain with complete discontinuation of tizanidine. The ultimate decision to transition the patient to methocarbamol and stop taking tizanidine for pain control highlights the importance of individualized care. The patient has responded to this therapy upon follow-up.

## Introduction

Tizanidine is a widely used imidazole-derived medication for spasticity and chronic pain [1]. It was approved by the FDA in 1996, and it is estimated that in 2021, there will be around 8 million active prescriptions of this medication in the United States [2]. Tizanidine is an alpha-2 agonist at the presynaptic level that works by modulating neurotransmitters (mainly GABA and glutamate), resulting in central analgesia [1], and acting at the imidazoline receptors with inhibition of spinal reflexes and improved spasticity [3].

Due to being a neurotransmitter modulator, tizanidine must be used with caution, as it has the potential for withdrawal [4]. Case reports in the literature suggest that withdrawal from tizanidine is possible. Abruptly stopping this medication, known for its inhibitory effect on the nervous system, can trigger a cascade of catecholamines, leading to symptoms such as hypertension, tachycardia, and spasticity [3-9]. Treatment involves restarting and subsequently de-escalating tizanidine, but patients in withdrawal must be treated using a conservative approach, as overtreatment or restarting tizanidine quickly at high doses can lead to an overdose with catastrophic hemodynamic outcomes like severe and persistent hypotension and bradycardia [10]. There is no standard of care for tizanidine withdrawal. Case reports show that withdrawals often respond to medications such as clonidine, lorazepam, and slowly restarting tizanidine [7,11-13]. To our knowledge, other neurotransmitter modulators have not been studied in the setting of withdrawal and overdose.

Without further ado, we present the case of a 68-year-old woman with chronic pain managed with opioids and tizanidine who arrived at the emergency department hypertensive, tachycardic, and reporting that she had abruptly stopped taking tizanidine, prompting suspicion of withdrawal from this agent.

## Case presentation

A 68-year-old woman with a history of chronic pain (managed with opioids and tizanidine 4 mg three times a day), hypertension (on lisinopril), COPD (on 2L of oxygen at home), thrombocytopenia, diastolic heart failure, panniculitis, and a previous osteomyelitis episode in her right lower extremity, was brought in from a facility after a ground level fall, as well as tachycardia, hypertension, tachypnea, nausea, and vomiting. On admission, the patient reported that she had not been taking her tizanidine daily as prescribed. The patient's presenting vital signs can be visualized in Table 1, and the laboratory tests done on admission can be found in Table 2.

**Table 1:** Comparison of the patient's vital signs on admission with the vital signs upon discharge

Variable	On admission	Upon discharge	Reference Range
Temperature (in degrees Celsius)	36.7	36.7	36.5-37.3
Respiratory Rate (respirations per minute)	30	16	12-20
Peripheral Pulse (beats per minute)	115	87	60-100
Systolic Blood Pressure (mm of Hg)	224	106	90-120
Diastolic Blood Pressure (mm of Hg)	193	88	60-80
Oxygen saturation at baseline O_2 _of 2L (%)	98	95	90-100

**Table 2:** Comparison of the patient's laboratory tests on admission with the laboratory tests upon discharge

Variable	On admission	Upon discharge	Reference Range
Sodium (mg/dL)	137	137	135-145
Potassium (mg/dL)	4.9	3.9	3.5-5
Chloride (mmol/L)	106	105	97-106
CO_2_ (mmol/L)	23	24	23-29
Blood Urea Nitrogen (mg/dL)	17	9	6-20
Creatinine (mg/dL)	0.87	0.75	0.7-1.2
Random Glucose (mg/dL)	107	87	64-100
Anion Gap (mEq/L)	11	8	4-12
Hemoglobin (g/dL)	15	15	12-16
Hematocrit (%)	46	46	36-48
White Blood Cell Count (× 10^9^/L)	6.3	4.8	4.5-11
Platelet number (per mcL)	283,000	280,000	150,000-450,000

**Figure 1:**
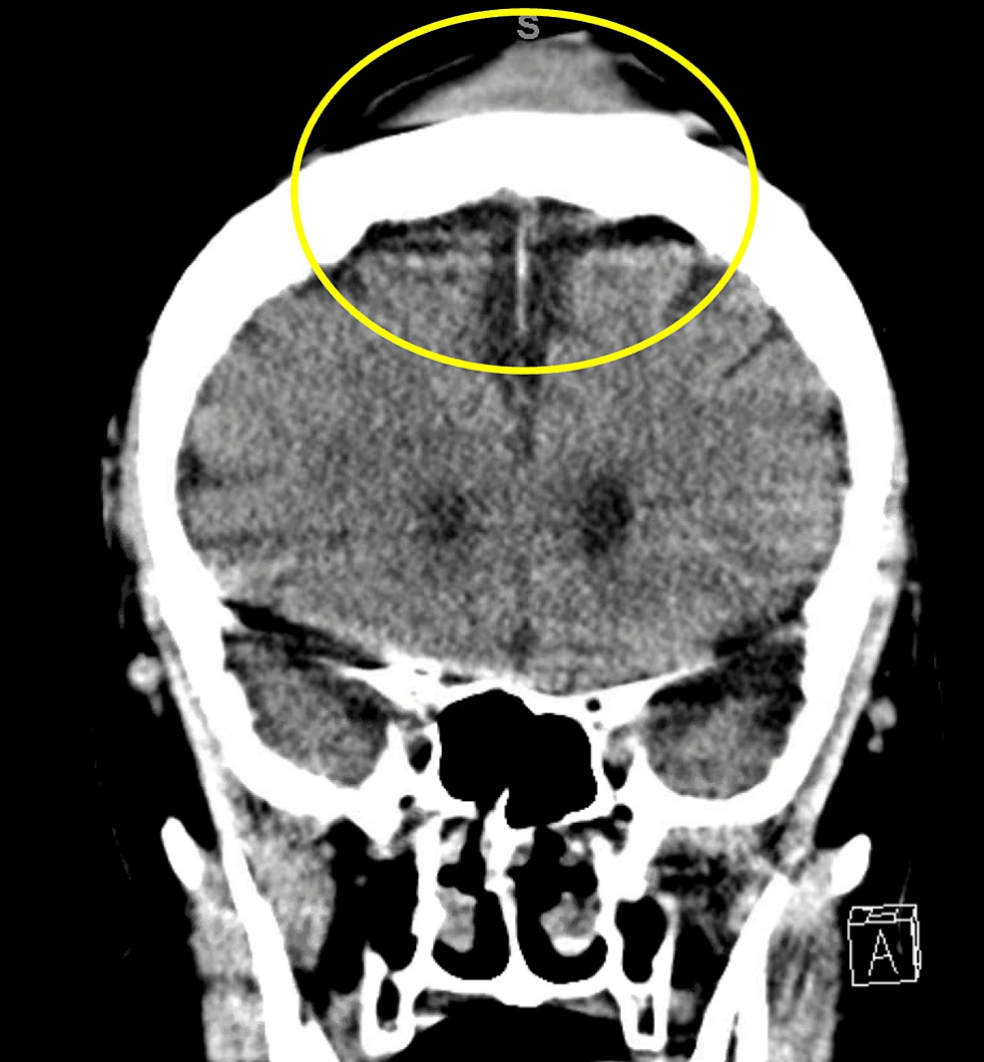
CT of the head The yellow circle highlights the area in which a vertex hematoma can be visualized, likely due to the level-ground fall that the patient sustained. There are no other abnormalities in this study.

In the emergency department, the patient’s hypertension and tachycardia were resolved with the initiation of her home tizanidine dose of 8 mg three times per day. On the other hand, diagnostic imaging done in the setting of the patient’s fall, including CT scans of the head, CT of the spine, and the abdomen/pelvis, showed no acute abnormalities except for a scalp hematoma that can be visualized in Figure 1.

The patient was admitted for tizanidine withdrawal. During the de-escalation of the tizanidine dose, especially when she was reduced to twice a day, she experienced withdrawal symptoms of tachycardia, hypertension, and severe pain. The patient received one dose of phenobarbital, considering its effects on glutamate, which she seemed to tolerate well. The resolution of the withdrawal symptoms demonstrates that phenobarbital can aid in tizanidine withdrawal. Vital signs after treatment and laboratory test results upon discharge can be visualized in Tables 1-2.

Despite the above complexities, the patient's condition was resolved, prompting consideration for discharge to a skilled nursing facility (SNF). The patient was given instructions to stop tizanidine and replace it with methocarbamol, a central-acting muscle relaxant, which she had tolerated in the past and had helped control her chronic pain along with her home opioids.

## Discussion

**Figure 2:**
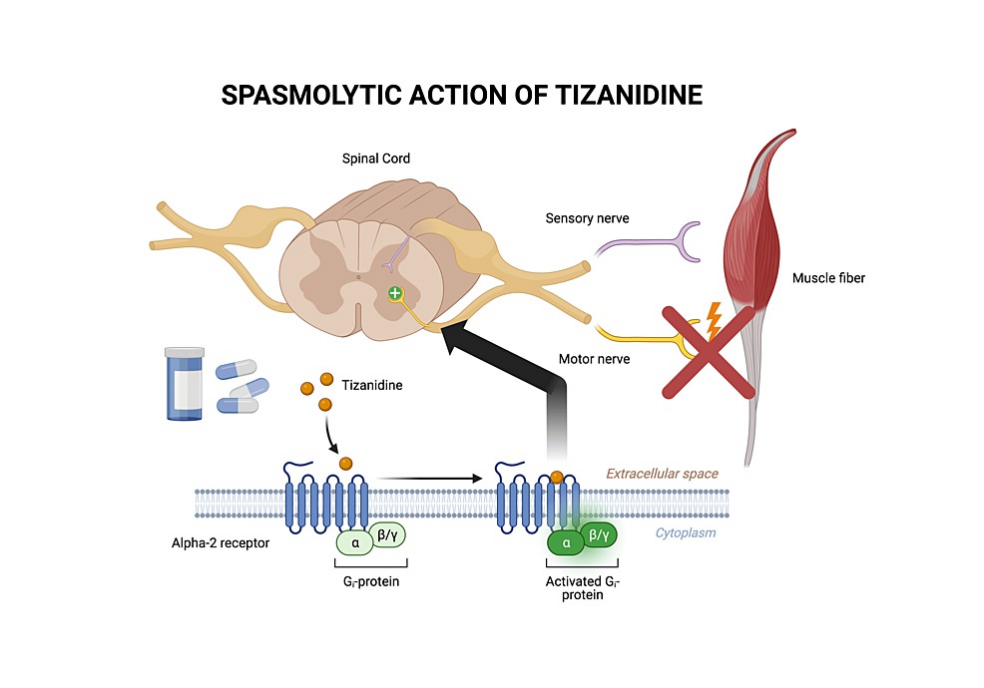
Mechanism of action of tizanidine Diagram depicting the spasmolytic action of tizanidine. The diagram is of the author’s own creation.

The presented case of tizanidine withdrawal in a 68-year-old woman explores the challenges of managing complications associated with this widely prescribed medication. Tizanidine, an alpha-2 agonist with dual actions on presynaptic neurotransmitters and imidazoline receptors, has been associated with withdrawal symptoms characterized by a cascade of catecholamines, leading to hypertension, tachycardia, and spasticity. The mechanism of action of tizanidine is illustrated in Figure 2.

Several case reports have shown that tizanidine withdrawal is possible, and they have resolved this complication by various means. The available case reports that we could find about tizanidine withdrawal are compiled in Table 3. One notable aspect of this case is the utilization of phenobarbital in managing the patient's altered hemodynamics. Phenobarbital, a barbiturate, acts as a positive allosteric modulator of the gamma-aminobutyric acid (GABA) receptor, leading to enhanced inhibitory neurotransmission. While tizanidine's primary mechanism of action involves the modulation of GABA and glutamate, the introduction of phenobarbital may have contributed to the resolution of symptoms through its GABAergic effects.

**Table 3:** Case reports available in the literature depicting tizanidine withdrawal

CASE REPORTS DEPICTING TIZANIDINE WITHDRAWAL				
Author	Year	Case	Strategy implemented	Outcome of the case
Morgom et al. [3]	2023	Tizanidine withdrawal	Tizanidine taper	Symptoms improved after initiating tizanidine taper
Suelt et al. [4]	2021	Tizanidine withdrawal	Tizanidine taper	Symptoms improved after initiating tizanidine taper, blood pressure normalized, and agitation stabilized
Perez et al. [5]	2009	Tizanidine withdrawal	Tizanidine taper	Symptoms improved after initiating tizanidine taper, blood pressure normalized, and agitation stabilized
Mörkl et al. [6]	2015	Tizanidine withdrawal and Takotsubo cardiomyopathy	Tizanidine taper	Symptoms improved after initiating tizanidine taper
Kitta et al. [7]	2020	Tizanidine withdrawal	Introduction of clonidine with subsequent tizanidine taper	After establishing clonidine, the off-tapering of tizanidine was much better tolerated by the patient and finally successful
Karol et al. [8]	2011	Baclofen and Tizanidine withdrawal	Slow taper of tizanidine and baclofen	Delirium and motor disturbances resolved within 24 h of reintroduction of medications
Daniels et al. [9]	2022	Tizanidine withdrawal	Tizanidine taper	A plan was made to attempt weaning her by 1 mg per dose weekly in the community

The enhanced GABAergic activity induced by phenobarbital could have mitigated the excessive catecholamine release associated with tizanidine withdrawal [14]. GABA, the principal inhibitory neurotransmitter in the central nervous system, plays a crucial role in regulating sympathetic outflow [14]. By potentiating GABAergic transmission, phenobarbital may have exerted a calming effect on the hyperactive sympathetic response triggered by tizanidine discontinuation, thereby alleviating the patient's hypertensive and tachycardic state [14].

This unique therapeutic approach with phenobarbital offers insights into potential strategies for managing tizanidine withdrawal, emphasizing the importance of understanding the interplay of neurotransmitter systems in the context of withdrawal syndromes. However, it is crucial to note that further research is needed to establish the safety and efficacy of phenobarbital in this specific clinical scenario.

The decision to transition the patient to methocarbamol for pain management also warrants consideration. Methocarbamol, a muscle relaxant with a distinct mechanism of action from tizanidine, was chosen to ensure adequate pain control while minimizing the risk of adverse events. This approach aligns with the principle of individualized care, recognizing the need to tailor treatment strategies to the unique characteristics and tolerances of each patient.

## Conclusions

In conclusion, managing tizanidine withdrawal requires a nuanced understanding of the pharmacological interactions involved. The use of phenobarbital in this case provides a compelling avenue for exploration, suggesting that agents affecting GABAergic neurotransmission may play a role in mitigating the hemodynamic consequences of tizanidine withdrawal. Further research is essential to validate and refine these approaches, paving the way for evidence-based guidelines in managing tizanidine-related complications.
